# Structure and Texture Synergies in Fused Deposition Modeling (FDM) Polymers: A Comparative Evaluation of Tribological and Mechanical Properties

**DOI:** 10.3390/polym17152159

**Published:** 2025-08-07

**Authors:** Patricia Isabela Brăileanu, Marius-Teodor Mocanu, Tiberiu Gabriel Dobrescu, Nicoleta Elisabeta Pascu, Dan Dobrotă

**Affiliations:** 1 Department of Robotics and Manufacturing Systems, Faculty of Industrial Engineering and Robotics, National University of Science and Technology POLITEHNICA Bucharest, 060042 Bucharest, Romania; patricia.braileanu@upb.ro (P.I.B.); tiberiu.dobrescu@upb.ro (T.G.D.); nicoleta.pascu@upb.ro (N.E.P.); 2 National Institute for Laser, Plasma and Radiation Physics Romania, 077125 Măgurele, Romania; contact@romocanu.ro; 3 Doctoral School of Industrial Engineering and Robotics, National University of Science and Technology POLITEHNICA Bucharest, 060042 Bucharest, Romania; 4 Faculty of Engineering, Lucian Blaga University of Sibiu, 550024 Sibiu, Romania

**Keywords:** 3D printing, polylactic acid, polymer composites load bearing, Ball-on-Disc testing, profilometry, Shore hardness, infill density, wear resistance, friction coefficient

## Abstract

This study investigates the interplay between infill structure and surface texture in Fused Deposition Modeling (FDM)-printed polymer specimens and their combined influence on tribological and mechanical performance. Unlike previous works that focus on single-variable analysis, this work offers a comparative evaluation of Shore D hardness and coefficient of friction (COF) for PLA and Iglidur materials, incorporating diverse infill patterns. The results reveal that specific combinations (e.g., grid infill with 90% density) optimize hardness and minimize friction, offering practical insights for design optimization in functional parts. Our aim is to provide design insights for enhanced wear resistance and hardness through tailored structural configurations. Carbon Fiber-reinforced PLA (PLA CF), aramid fiber-reinforced Acrylonitrile Styrene Acrylate (Kevlar), and Iglidur I180-BL tribofilament. Disc specimens were fabricated with gyroid infill densities ranging from 10% to 100%. Experimental methodologies included Ball-on-Disc tests conducted under dry sliding conditions (5 N normal load, 150 mm/s sliding speed) to assess friction and wear characteristics. These tribological evaluations were complemented by profilometric and microscopic analyses and Shore D hardness testing. The results show that Iglidur I180-BL achieved the lowest friction coefficients (0.141–0.190) and negligible wear, while PLA specimens with 90% infill demonstrated a polishing-type wear with minimal material loss and a friction coefficient (COF) of ~0.108. In contrast, PLA CF and Kevlar exhibited higher wear depths (up to 154 µm for Kevlar) and abrasive mechanisms due to fiber detachment. Shore hardness values increased with infill density, with PLA reaching a maximum of 82.7 Shore D. These findings highlight the critical interplay between infill architecture and surface patterning and offer actionable guidelines for the functional design of durable FDM components in load-bearing or sliding applications.

## 1. Introduction

The recent advancements in additive manufacturing (AM) technologies, particularly in Fused Deposition Modelling (FDM), have significantly transformed the fields of industrial design and fabrication. These developments have enabled the production of complex geometries and high-performance components tailored for diverse sectors, including aerospace, robotics, automotive engineering, and biomedical applications [1,2,3,4,5]. The widespread adoption of FDM is largely attributed to its cost-efficiency, operational simplicity, and intrinsic versatility in processing various thermoplastic materials [4,5,6].

As highlighted in the study by Batista et al. [4], a key factor in enhancing the functional performance of 3D-printed parts lies in the precise control of both their internal mesostructure (infill geometry) and external surface morphology. Such architectural manipulation allows for functional customization of components, thereby improving their mechanical, tribological, and thermal properties according to the requirements of specific applications.

The theoretical foundation of this work is grounded in the core principles of additive manufacturing as articulated by Gibson et al. [1], who emphasized the critical role of process parameters, internal infill patterns, and surface topography in determining part performance. FDM, in particular, relies on a layer-by-layer material deposition approach, which inherently induces anisotropic mechanical properties and interlayer adhesion weaknesses. However, these limitations can be mitigated through deliberate architectural optimization, including infill orientation strategies and layer height adjustments [1,2].

Furthermore, foundational studies in tribology by Rabinowicz [3] and later by Bhushan [5] underscored the influence of contact mechanics, surface energy, and interfacial phenomena on wear and frictional behavior. These tribological principles become especially relevant in polymeric systems with complex surface textures, such as those produced via FDM, where surface roughness and topographic features significantly affect performance under dynamic loading and sliding conditions.

Internal infill architectures, such as the gyroid, play a fundamental role in enhancing the mechanical performance and material efficiency of FDM-fabricated components [6,7,8,9]. These three-dimensional structures are specifically engineered to promote homogenous, quasi-isotropic mechanical properties, thereby increasing strength in multiple loading directions while simultaneously enabling weight reduction [2,10]. Infill density has been shown to directly influence structural integrity, as higher densities reduce void content, improve load transfer, and enhance both tensile and yield strength [1]. Moreover, infill patterns like honeycomb and gyroid have demonstrated superior mechanical behavior due to their characteristic layer orientation, which contributes to effective crack retardation and damage distribution under stress [11].

Beyond internal geometry, the surface characteristics of FDM-printed parts—particularly surface roughness and topographical texture—have a decisive influence on their tribological performance [1,7]. Friction, a dominant contributor to wear and energy dissipation in mechanical systems, represents a substantial share of global energy losses, emphasizing the urgency of developing materials and design strategies that mitigate its effects [12]. While smoother surfaces generally reduce friction and wear rates, strategically textured surfaces have been shown to improve tribological outcomes by facilitating lubrication and trapping wear debris [1]. Experimental findings suggest that specific surface textures can significantly lower the coefficient of friction (COF) in dry sliding conditions [1,12]. For instance, FDM-fabricated polylactic acid (PLA) samples with engineered surface texturing exhibit reduced COF values, especially when using cubic or symmetric geometries, which perform better tribologically than anisotropic or biomimetic patterns [1,12].

The intrinsic material properties of the polymers used also play a pivotal role in tribological behavior. For example, the amorphous microstructure of polyethylene terephthalate glycol (PETG) tends to yield lower COF values, occasionally approaching superlubricity, in contrast to more crystalline structures like PLA [12,13]. Conversely, the integration of reinforcements—such as randomly dispersed carbon fibers—may elevate friction levels due to increased surface asperities and microstructural heterogeneity [13].

Extensive investigations have addressed the tribological responses of a wide range of FDM-compatible polymers and composites under sliding conditions [1,13,14,15,16,17]. Among these, polyamide (PA) and copper-epoxy composites have demonstrated excellent wear resistance, with PA exhibiting intrinsic lubricity and effective counterface interactions, particularly when paired with low-friction alumina [18]. In contrast, fiber-reinforced PLA composites generally exhibit higher wear rates than their unfilled counterparts. This behavior is attributed to microstructural imperfections, such as fiber misalignment and voids at the fiber–matrix interface, which significantly increase surface roughness—by up to 35–40%—and thereby accelerate material degradation [18].

While the incorporation of solid lubricants has proven more effective than traditional reinforcements in improving wear resistance [18], carbonaceous fillers—such as short carbon fibers (SCFs) and graphene nanoplatelets (GNPs)—have shown promise in enhancing mechanical properties and specific wear resistance [3,18]. For instance, PLA composites containing 5 wt.% SCF and 5 wt.% GNP has demonstrated up to a 30% increase in hardness, a 220% increase in elastic modulus, and a fivefold reduction in specific wear rate, accompanied by significantly lower COF values [3]. However, higher filler concentrations, particularly of SCF, can lead to more rigid composites with increased frictional behavior due to rougher surfaces [3].

Metallic particle reinforcements, such as copper or aluminum, have also been explored for their beneficial effects. PLA composites with aluminum additions have shown increases in material hardness exceeding 75% and a 50% reduction in wear volume, albeit with a potential trade-off in terms of increased surface roughness and friction [4,19]. Moreover, post-processing techniques aimed at improving surface smoothness may inadvertently compromise mechanical toughness and structural integrity of the final printed parts [20].

Despite the expanding body of research on FDM-fabricated polymers, a significant knowledge gap remains regarding the integrated analysis of internal infill architectures and external surface texturing within a unified experimental framework [2,8,21]. Current studies tend to isolate these variables, either focusing on mechanical strength optimization or tribological enhancement, rarely addressing their combined influence on performance under dynamic, high-stress applications [1,3,22]. A more holistic approach is therefore required to understand the synergistic interplay between mesostructural design and surface engineering for advanced functional applications.

Recent reviews on continuous-fiber FDM composites [22] also highlight the importance of infill design and fiber orientation in achieving optimal mechanical and tribological performance.

This study aims to bridge this gap by providing a comparative evaluation of how internal design parameters, specifically gyroid pattern, infill density, and external surface characteristics, exemplified by Archimedean pattern texturing, synergistically influence the tribological and mechanical responses of FDM-printed components of different materials. Systematically investigating key performance indicators, including surface wear, the coefficient of friction, and Shore hardness across a range of four polymeric materials (generic PLA, PLA CF, Kevlar, and Iglidur I180-BL tribofilament). Thus, the findings may offer actionable insights for designing high-performance FDM polymer parts by leveraging the interplay between their structured internal architecture and engineered external surfaces.

## 2. Materials and Methods

The selection of polymeric materials for this comparative tribological and mechanical study of FDM-printed specimens was strategically made to encompass a diverse range of properties critical for additive manufacturing applications, thereby enabling comprehensive parametric analysis under varying infill and surface texture conditions. Generic polylactic acid (generic PLA filament manufactured by INNOVATEFIL^®^, Smart Materials 3D, Alcalá la Real, Jaén, Spain) was included as a foundational material due to its widespread compatibility with fused deposition modeling 3D printers, ease of processing characterized by minimal shrinkage, and ability to yield a good surface finish [19], with documented mechanical properties such as a tensile strength at break of 53 MPa and a flexural strength of 83 MPa [23].

To investigate the impact of reinforcement, polylactic acid reinforced with carbon fibers (PolyLite^™^ PLA-CF filament manufactured by Polymaker^®^, Shanghai, China) was chosen for its significantly enhanced rigidity and mechanical properties, including a Young’s modulus of 2945 ± 100 MPa (X-Y) and a Charpy impact strength of 4.82 ± 0.14 kJ/m^2^, which, combined with its characteristic smooth and matte surface finish, positions it as a robust FDM material [24].

The Kevlar composite (ApolloX Kevlar filament manufactured by FormFutura^®^ 3D Printing Materials, Gelderland, The Netherlands), an aramid fiber-reinforced ASA filament (Acrylonitrile Styrene Acrylate), was selected for its distinct shatterproof characteristics, greatly improved impact and damage resistance, and ability to mask layer lines with a matte surface finish, offering a contrasting performance profile to the PLA-based materials, despite its abrasive nature necessitating hardened steel or ruby nozzles [25]. Table 1 presents a comparative overview of the principal mechanical and physical properties of the four filaments selected for this study, highlighting differences in strength, stiffness, elongation, and density that justify their inclusion in the experimental evaluation of structure and texture synergies in FDM-printed components.

Iglidur I180-BL (tribofilament manufactured by igus^®^ GmbH, Cologne, Germany) was incorporated to specifically evaluate tribological performance, being an engineered thermoplastic compound designed for moving applications, exhibiting superior wear resistance (up to 50–100 times greater than standard filaments) and an exceptionally low coefficient of friction (µ = 0.15–0.25) without external lubrication [26,27], with FDM compatibility requiring nozzle temperatures of 250–260 °C and an enclosed build volume for optimal part performance [26,27].

All average values of the coefficient of friction (COF) and wear depth are reported alongside their corresponding minimum and maximum values in the results section. While standard deviations (±) are not shown directly in all cases, the data were obtained from triplicate tests (n = 3) for each configuration to ensure statistical reliability. Where relevant, standard deviations are available upon request and were used to verify the consistency of trends.

### 2.1. Specimen Fabrication and Preparation Protocol

**Figure 1:**
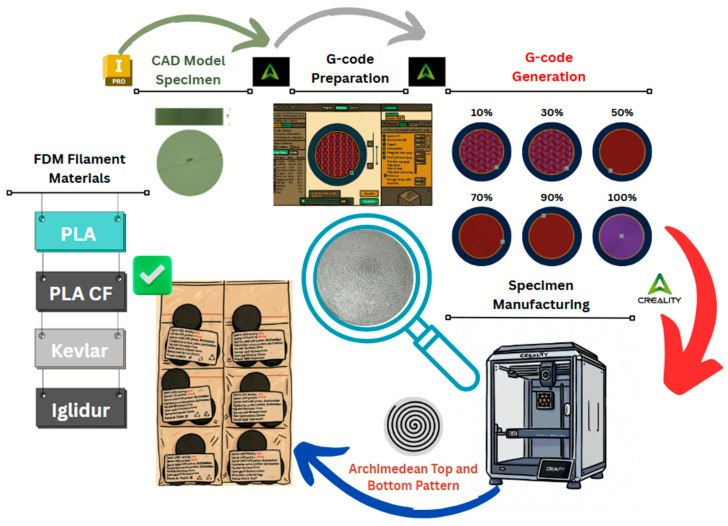
Overview of the specimen preparation process used in this study.

Since the specimens were used for Ball-on-Disc testing in the initial phase of the experiments and considering the specific mounting system of the tribometer employed in this study, disc-shaped specimens were selected to ensure proper fixation and handling. The preparation process began with CAD modeling, where a 3D model of a cylinder with a diameter of 40 mm and a height of 10 mm was created. The model was then exported in STL format to generate the corresponding G-code. The STL file was subsequently imported into Creality Print 6.0 slicing software. Figure 1 provides an overview of the design preparation process and 3D printing specimens used in this study.

To arrive at an optimal interlayer adhesion and consistent results across materials with varying thermal and rheological properties, the extrusion temperature, bed temperature, and printhead movement speeds were individually optimized for each polymer, as summarized in Table 2.

All specimens were fabricated using a Creality K1C FDM printer (Creality Co., Ltd., Shenzhen, China) equipped with an all-metal direct-drive extruder and a 1.75 mm hardened steel nozzle, which allows for high-speed printing of fiber-reinforced materials such as PLA CF while maintaining high layer resolution. The printer’s enclosed build chamber helped maintain stable ambient temperature and humidity throughout the fabrication process, thereby preventing common printing defects such as thermal deformation, delamination, or interlayer instability. Moreover, since the materials used in this study are hygroscopic and prone to moisture absorption from the environment, all filaments were pre-dried using the Creality Space Pi Filament Dryer (Creality Co., Ltd., Shenzhen, China). This step was critical for preventing the formation of bubbles, delamination, or uncontrolled porosity during printing and to ensure the validity of the experimental data.

In order to obtain relevant comparative results, all specimens were fabricated with identical geometrical parameters. The general slicing settings included 2 perimeter walls, 4 solid top and bottom layers, an Archimedean pattern applied to both the top and bottom surfaces, a gyroid sparse infill structure, and a standard layer height of 0.20 mm. To investigate the influence of internal structure on mechanical behavior and identify any correlation between infill density and mechanical response, G-code files were generated for six different sparse infill densities: 10%, 30%, 50%, 70%, 90%, and 100%. For the 100% infill configuration, all layers were printed with the Archimedean pattern, resulting in a fully solid specimen. Three specimens were printed for each material and configuration in order to ensure minimum statistical reproducibility of the experimental results.

All specimens were fabricated under controlled environmental conditions, with a room temperature of 24 ± 1 °C and a relative humidity of approximately 50%, to minimize degradation and dimensional instability during fabrication.

To improve bed adhesion during additive manufacturing, a circular skirt was generated around the base of each cylinder. No adhesion problems were observed for PLA, PLA CF, or ApolloX Kevlar. However, adhesion issues occurred when printing Iglidur I180-BL (igus^®^ GmbH, Cologne, Germany) specimens, which were resolved by applying a 3D LAC adhesive (3DLAC^®^, Zamora, Spain) to enhance the bond between the specimen and the heated bed.

After the fabrication process was completed, all specimens were post-processed to remove the skirt. Surface dust and microparticles were removed using compressed air, and the specimens were cleaned with Kimwipes^®^ (Kimberly-Clark Corporation, Irving, TX, USA) and 99% isopropyl alcohol- IPA (Kynita S.R.L., Budești (Răcovița), Vâlcea, Romania) to eliminate any surface grease residues.

### 2.2. Ball-on-Disc Experiment Preparation

**Figure 2:**
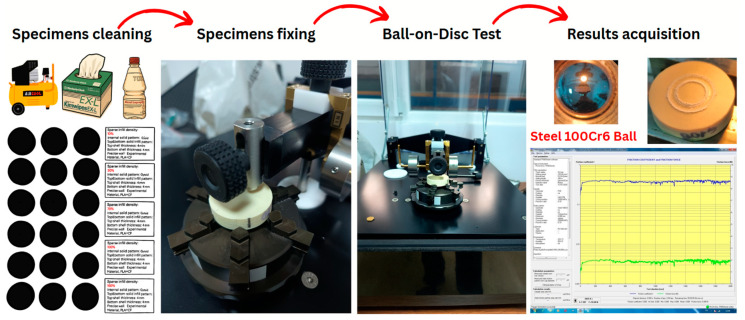
Specimen preparation for the Ball-on-Disc wear testing.

To conduct Ball-on-Disc tribological experiments, the TRIBOtester system (manufactured by Tribotechnic US LLC, San Francisco, CA, U.S.A.) was used, as shown in Figure 2. The experimental protocol was designed to provide a comprehensive, general description of the experimental methodology, ensuring reproducibility and consistency across the material samples selected for this study.

The tribological tests followed the well-established ASTM G99-17 standard [28], which provides a reproducible framework for measuring wear and friction in pin-on-disk or Ball-on-Disc configurations. This method has been foundational for decades in tribology and was originally designed to simulate adhesive, abrasive, or mild oxidative wear regimes under controlled contact mechanics [3,28].

All tribological tests were performed using a Ball-on-Disc configuration using the ASTM G99 standard, where the material specimens function as the rotating disc and the steel ball (Tribotechnic US LLC, San Francisco, CA, U.S.A.) serves as the stationary pin. This arrangement indicates a vertical application of the normal load onto the horizontal surface of the rotating disc. The Ball-on-Disc tribological tests were performed under ambient conditions and dry sliding contact, where no lubricant was introduced at the interface between the polymer specimens and the steel ball.

The rotating friction counterbody was a spherical ball manufactured from Steel 100Cr6 (according to DIN 17230 standard) [29]. The ball used in this research had a nominal diameter of 6000 µm (6 mm) and was characterized by a Young’s modulus of 205 GPa, with a Poisson’s ratio typically of 0.33.

All specimens’ surfaces were blown free of dust and microparticles using compressed air and cleaned with IPA-soaked lint-free wipes, after allowing the surfaces to dry in a clean environment, prior to the experimental tests.

The tribological tests were consistently performed in a controlled laboratory environment. The ambient temperature was maintained at 24 ± 1 °C, and the relative humidity was set around 50%. All measurements were conducted under an air atmosphere.

The Ball-on-Disc tests were executed with a standard normal load of 5 N, although the TRIBOtester system supports an extended load range. The sliding speed for all tests was consistently set at 150 mm/s, and each experiment was conducted for a total sliding length of 300 m. The Hertz pressure was calculated to be 231 MPa for a 5 N load.

The steel ball was mounted in a fixed position, maintaining perpendicularity to the rotating planar surface of the material specimens. The normal load was applied vertically, ensuring consistent contact between the ball and the specimens throughout the test duration. This standard alignment facilitates the formation of a circular wear track on the specimens and a corresponding wear cap on the spherical ball, which were subsequently documented through post-test microscopical imaging and profilometry.

During each tribological test, the TRIBOtester system acquired real-time data on the friction force and friction coefficient. The reported friction coefficient values included the initial, average, minimum, and maximum coefficients of friction for each material studied. Wear characteristics were primarily evaluated through visual inspection and subsequent microscopic analysis and profilometry, with consistent imaging documentation generated throughout the process.

### 2.3. Profilometric Assessment of Wear Tracks

**Figure 3:**
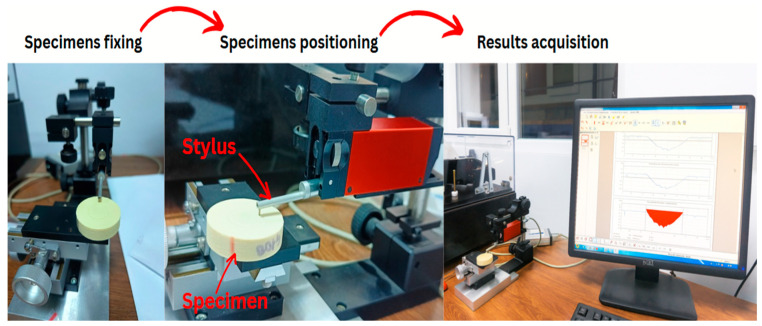
Specimen preparation and profilometric analysis sequence.

Surface profilometry characterization was systematically performed on all tested specimens that showed signs of wear. The profilometric analysis was conducted using a stylus profilometer manufactured by Tribotechnic US LLC (San Francisco, CA, USA). The profilometric characterization tests were consistently performed in a controlled laboratory environment. The ambient temperature was maintained at 24 ± 1 °C, and the relative humidity was set to 50%. All measurements were conducted under an air atmosphere. The specimens were secured in the profilometer’s clamping system, and the stylus was positioned over the area of interest on the surface to enable relevant measurements, as shown in Figure 3

For each specimen, multiple linear profilometric scans were conducted along a defined length, specifically 4 mm for all materials analyzed. To ensure data reliability and account for localized variations, a series of three independent scans was performed for all specimens, and the resulting measurements were subsequently averaged for parameters such as maximum depth, area of the hole, maximum height, and area outside. Qualitative observations regarding the visibility of the profile and the presence of traces on the steel ball were also recorded. The extracted parameters followed the ISO 4287 [30] amplitude parameters for the profiles. All parameters were consistently processed using a Gaussian filter with a cut-off wavelength of 0.8 mm.

All standard deviation values have been rounded in accordance with the resolution limit of the profilometric equipment (±1 µm), to ensure consistency between numerical precision and measurement capabilities.

For a more comprehensive understanding of deposition mechanisms in FDM processes, particularly in relation to reinforcement patterns and structural behavior, the review by He F. et al. provides a thorough classification and analysis of continuous-fiber-reinforced composites [31].

### 2.4. Microscopic Analysis

**Figure 4:**
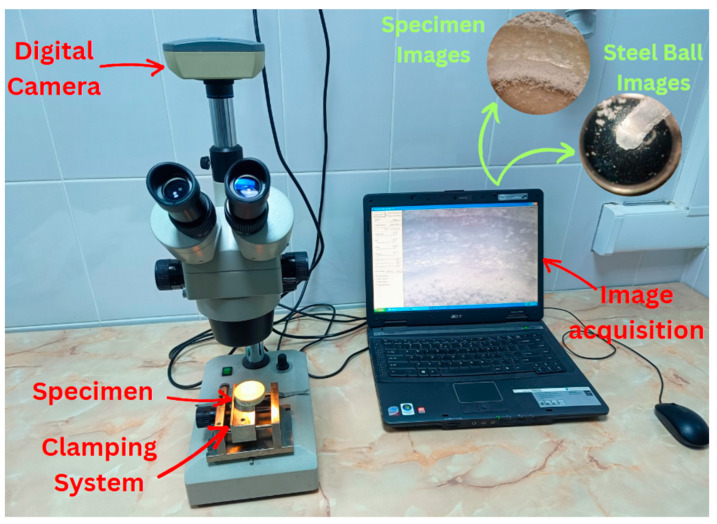
Specimen preparation for analysis of the worn surfaces.

To analyze the wear surfaces of the specimens, a digital stereo optical microscope (NOVEX RZT-SF, Euromex, The Netherlands) equipped with a CMEX DC.1300x USB camera (Euromex Microscopen B. V., Duiven, The Netherlands) and ImageFocus v 2.5 software was used, enabling three-dimensional observation of the wear tracks left by the steel ball on the material surfaces. Each specimen was placed on the stage of the digital microscope and secured in an adjustable mechanical holder to prevent any displacement during focusing. Images were captured at a total magnification of 20× (objective 10× combined with a 2× magnification base). The microscope is equipped with built-in lighting, oriented to highlight the surface details of the specimens, as well as with a digital camera that allows real-time visualization and image acquisition directly on the computer, as shown in Figure 4.

Surface images were collected at various magnification levels, allowing the evaluation of the general surface appearance both before and after the Ball-on-Disc test, to observe the wear tracks or any damage resulting from the tribological test.

### 2.5. Shore Hardness Evaluation

The hardness of the polymer specimens was evaluated using a Shore D durometer manufactured by Shenzhen Rongbo Jiachuang Technology Co., Ltd. (Shenzhen, China) (0–100 HD range, resolution of 0.5 HD units), in accordance with standardized procedures for rigid plastic materials (GB/T531.1-2008 standard [32], equivalent to ISO 868 [33]/ASTM D2240 [34]). Each specimen in this study was tested six times to ensure statistical consistency of the results. The indenter was applied perpendicularly to the specimen surface under a controlled load, and the hardness value was recorded after a dwell time of 3 s. Depending on the stiffness of the material analyzed, different values were obtained and compared. All measurements were conducted under controlled ambient conditions (24 ± 1 °C and 50% relative humidity) and the results were reported as average values followed by the corresponding standard deviation.

The experimental methodology employed across all phases of this study was thoughtfully planned to ensure comprehensive methodological consistency, precise experimental oversight, and high reproducibility, thereby facilitating valid comparative analyses among the diverse FDM filaments investigated. All specimen fabrication, hardness testing, profilometry, and Ball-on-Disc tribological assessments adhered to a coherent experimental setup.

## 3. Results

The results obtained at each experimental stage were collected for all the materials studied and were first analyzed individually to determine the correlation between the experimentally obtained values and the specimens’ infill density. Afterward, a comparative analysis of the four materials studied was carried out.

To make the presentation of the experimental data clearer, arithmetic means of the obtained values were calculated using Equation (1). Average experimental values were calculated as the arithmetic means of the recorded values during the test:

(1) $$\bar{x}=\frac{1}{n}\sum_{i=1}^{n}x_{i},$$

where $\bar{x}$ is the arithmetic mean of the measurements, *x_i_* is the individual value of the measurement set, and *n* represents the total number of measurements performed during testing.

The standard deviation for each experimental set of measurements was calculated using the following formula:

(2) $$SD=\sqrt{\frac{1}{n-1}\sum_{i=1}^{n}{\left(x_{i}-\bar{x}\right)}^{2}},$$

where *SD* is the specimen standard deviation, *x_i_* represents each individual measurement, $\bar{x}$ is the arithmetic mean of the measurements, and *n* is the total number of measurements taken.

### 3.1. Ball-on-Disc Experimental Results

#### 3.1.1. Generic PLA Material

Generic PLA specimens exhibited varied tribological performance depending on the infill density. The friction coefficient generally ranged from 0.092 to 0.487. Particularly, the PLA with 90% infill specimen demonstrated significantly lower friction, with an average coefficient of 0.108 (values ranging from 0.092 to 0.139). In Table 3, the average values of COF can be viewed for each infill density.

Regarding wear, the PLA with 50% infill specimen developed a visible, relatively deep profile with white span stuck to the steel ball and slight scratches on the ball, as observed post-test. For generic PLA with 10%, 30%, 70%, and 100% infill density, the wear profile was visible but hard to discern under the microscope due to low contrast, characterized by long laminar chips and no trace on the 100Cr6 steel ball. The generic PLA with 90% infill density specimen, despite its low friction, showed only a slightly visible profile, described as fine polishing, with no steel ball traces or debris. This may suggest that higher infill percentages (specifically 90% infill density) can lead to a smoother wear mechanism, resulting in polishing rather than abrasive material removal.

**Figure 5:**
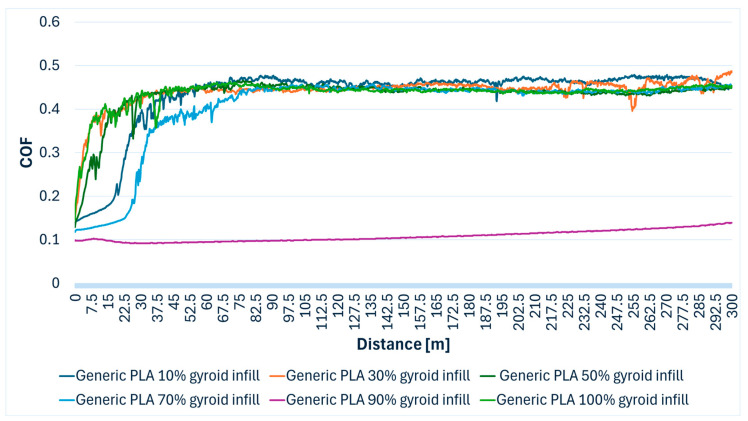
Generic PLA (polylactic acid) specimens’ COF variation during the Ball-on-Disc experiment.

Figure 5 illustrates the COF evolution over the sliding wear distance for generic PLA specimens fabricated with various gyroid infill densities. A rapid increase in COF is observed at the beginning of the test, followed by a stabilization phase, and the influence of infill density on frictional behavior is evident, with denser structures generally exhibiting more consistent performance.

#### 3.1.2. PLA with Carbon Fiber Material

PLA CF composites displayed a uniform friction coefficient ranging from 0.139 to 0.409 across all tested infill densities. In Table 4, the minimum and maximum COF values recorded for each infill density and the average value of COF can be viewed.

**Figure 6:**
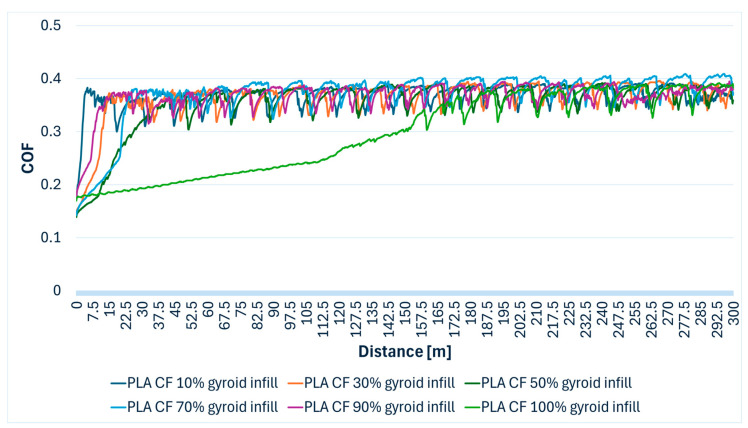
PLA CF (polylactic acid with carbon fiber) specimens’ COF variation during the Ball-on-Disc experiment.

Most PLA CF specimens (10%, 30%, 90%, and 100% infill densities) showed a visible profile and a trace on the steel ball, which indicates material removal and transfer to the 100Cr6 steel ball. This demonstrates that the carbon fiber reinforcement, while potentially enhancing mechanical properties, may contribute to an abrasive wear mechanism under dry sliding, where exposed fibers act as an abrasive third-body or cause micro-cutting. In Figure 6, the evolution of the COF in relation to the sliding distance for PLA CF specimens printed with various gyroid infill densities can be observed.

#### 3.1.3. ApolloX Kevlar Material

The ApolloX Kevlar filament exhibited average friction coefficients consistently around 0.126 to 0.456 across all infill densities. A defining characteristic of the ApolloX Kevlar filament was its significant wear behavior. In Table 5, the minimum and maximum COF values ecorded for each infill density and the average value of the friction coefficient can be viewed.

**Figure 7:**
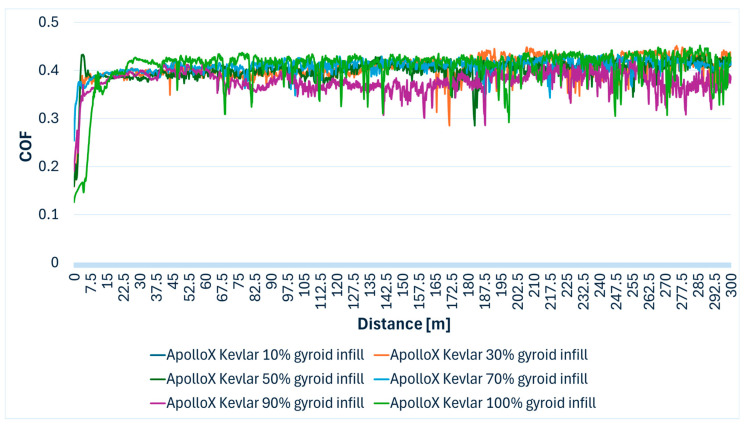
ApolloX Kevlar specimens COF variation during the Ball-on-Disc experiment.

Observations consistently reported a visible, deep profile wear track on the specimens, accompanied by a considerable amount of white wear debris and a trace on the test steel ball. This extensive wear and debris formation indicates a significant abrasive component to the tribological interaction, likely due to the hard Kevlar fibers detaching and acting as abrasive particles. Figure 7 shows variation in the COF over the 300 m wear distance for ApolloX Kevlar specimens manufactured with gyroid infill densities of 10% to 100%.

#### 3.1.4. Iglidur I180-BL Tribofilament

Iglidur I180-BL tribofilament demonstrated excellent tribological performance, characterized by very low friction coefficients and minimal wear across all tested infill percentages. The friction coefficient was consistently low, ranging from 0.116 to 0.431. In Table 6, the minimum and maximum COF values recorded for each infill density and the average value of the friction coefficient can be viewed.

**Figure 8:**
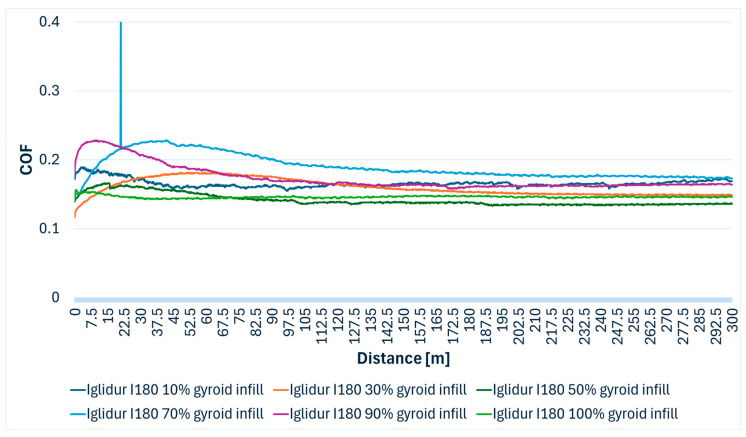
Iglidur I180-BL specimens’ COF variation during the Ball-on-Disc experiment.

Importantly, visual observations confirmed no discernible profile on the samples and no trace on the steel ball for all Iglidur I180-BL specimens. For the 10% infill specimen, fine detached grey threads were observed, which could represent very fine wear debris or material transfer. The superior tribological performance of Iglidur I180-BL aligns with the known self-lubricating properties of these tribofilament materials, which are engineered to reduce friction and wear through embedded solid lubricants or inherently low-friction polymer matrices. Figure 8 presents variations of the coefficient of friction over the wear distance for Iglidur I180-BL tribofilament specimens fabricated with different gyroid infill densities (from 10% to 100%).

### 3.2. Profilometry Results

The tribological performance of the polymeric specimens was quantitatively assessed through profilometric analysis of wear tracks generated during Ball-on-Disc tribological testing.

#### 3.2.1. PLA Generic Material

PLA specimens exhibited visible and relatively deep wear profiles, with some specimens leaving slight scratches or white polymeric span on the 100Cr6 steel ball. Table 7 shows the arithmetic means of the measurements obtained using the profilometer, from which it can be observed that the density percentage of the gyroid pattern significantly influences the appearance of wear marks.

Single profilometric measurements of all generic PLA specimens showed that the arithmetic mean roughness *Ra* ranged from 3.50 µm for 30% infill to 10.1 µm for 70% infill. The average peak-to-valley height *Rz* ranged from 20.8 µm for 30% infill to 55.8 µm for 70% infill. The total height of the profile *Rt* ranged from 31.8 µm for 30% infill to 68.3 µm for 70% infill and the root mean square roughness *Rq* ranged from 4.22 µm for 30% infill to 13.3 µm for 70% infill. The Skewness *Rsk* ranged from −0.767 for 70% infill density to −2.44 for 100% infill density, which confirms a predominance of valleys on the surface. Also, the Kurtosis *Rku* parameter ranged from 4.76 for 70% infill to 9.55 for 100% infill. As a result, PLA specimens showed varying degrees of surface degradation, with 30% infill density demonstrating generally lower average roughness parameters and maximum depth compared to higher infill densities. The observed increases in roughness at 50% and 70% gyroid infill density suggest that intermediate infill densities may lead to less optimal load distribution, resulting in greater surface deformation during wear.

#### 3.2.2. PLA CF Material

PLA CF material, chosen for its significantly enhanced rigidity and mechanical properties, generally demonstrated better wear resistance compared to generic PLA, although observations varied with infill density.

As shown in Table 8, the profilometric measurements of all PLA CF specimens showed that *Ra* ranged from 3.01 µm for 10% infill to 5.92 µm for 90% infill. The *Rz* ranged from 18.6 µm for 10% infill density to 34.9 µm for 70% infill density, while *Rt* varied from 30.5 µm for 10% infill to 49.5 µm for 70% infill. The *Rq* ranged from 3.91 µm for 10% infill to 7.77 µm for 70% infill. Also, the *Rsk* ranged from −0.893 for 10% infill to −2.34 for 100% infill and *Rku* values ranged from 3.96 for 70% infill density to 8.73 for 100% infill density, indicating surface profiles with sharp peak valley structures, particularly at higher infill densities.

The average lower maximum depths and overall reduced roughness parameters for PLA CF compared to generic PLA, especially at 10% and 100% infill density, emphasize the enhanced wear resistance conferred by carbon fiber reinforcement. The Archimedean pattern used for the solid top provided a robust initial contact surface combined with the reinforcing fibers, limiting in this way the severe wear.

#### 3.2.3. ApolloX Kevlar Material

The Kevlar specimens consistently exhibited a visible, deep profile, producing a large amount of white dust and leaving traces on the 100Cr6 steel ball. These observations can suggest a more abrasive wear mechanism, consistent with Kevlar’s abrasive nature. The measured wear depths and areas of the hole were notably larger than those for generic PLA and PLA CF materials as shown in Table 9.

Individual measurements of all ApolloX Kevlar composite specimens showed that *Ra* ranged from 3.52 µm for 70% infill to 6.04 µm for 90% infill. The average peak-to-valley height *Rz* varied between 18.1 µm for 50% infill and 36.2 µm for 90% infill, while the total height of the profile *Rt* ranged from 27.3 µm for 100% infill to 50.9 µm for 90% infill. The root mean square roughness *Rq* spanned from 4.42 µm for 30% infill to 7.88 µm for 90% infill. The skewness *Rsk* ranged from −0.966 for 30%infill to +0.187 for 100% infill. Also, the kurtosis *Rku* ranged from 2.68 for 100% infill to 7.48 for 30% infill, suggesting that some of the specimens exhibited sharply peaked surface features due to their heterogeneous microstructure and filler distribution.

Despite Kevlar’s shatterproof characteristics and greatly improved impact and damage resistance, its abrasive nature likely resulted in the observed significant material removal and wear track formation, as indicated by the consistently high maximum depth values across all infill densities.

The relatively consistent *Ra* values for Kevlar, despite large wear depths, suggest that while significant material is removed, the remaining worn surface maintains a comparable level of average roughness to PLA CF, though the overall topographical change (depth and area) is far more pronounced.

#### 3.2.4. Iglidur I180-BL Material

Iglidur I180-BL exhibited minimal to no discernible wear profiles after the Ball-on-Disc tribological test. Across all tested gyroid infill densities, they consistently showed no discernible profile and no trace on the 100Cr6 steel ball, with additional observations of insignificant detached grey powder for the 10% infill specimen. This outstanding wear resistance aligns with Iglidur I180-BL’s design as an engineered thermoplastic compound known for superior wear resistance and an exceptionally low COF.

### 3.3. Microscopic Results

**Figure 9:**
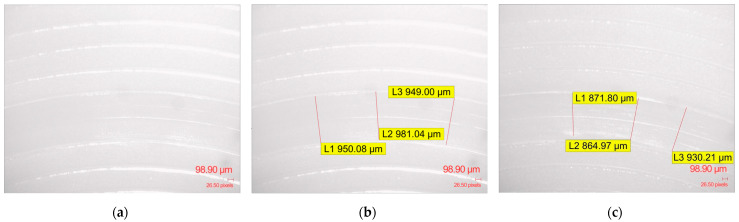
Microscopic evaluation of the wear track morphology surface after Ball-on-Disc testing of generic PLA material: (**a**) specimen with 10% gyroid pattern infill; (**b**) specimen with 10% gyroid pattern infill wear traces; (**c**) specimen with 100% gyroid pattern infill wear traces.

Among all investigated infill densities of the specimens analyzed in this study, the fundamental surface topography was consistently presented as a series of well-defined, albeit slightly irregular, parallel ridges and valleys (Figure 9a).

**Figure 10:**
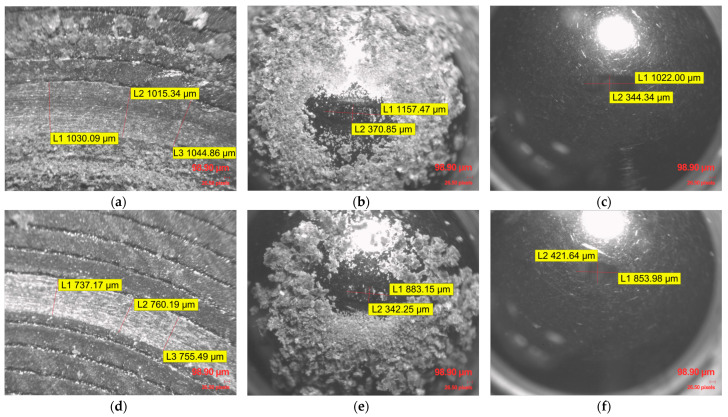
Microscopic evaluation of the wear track morphology and counterbody surface after Ball-on-Disc testing of PLA CF material: (**a**) PLA CF specimen with 30% gyroid pattern infill wear traces; (**b**) 100Cr6 steel ball covered with PLA CF debris after tribological testing of 30% infill specimen; (**c**) 100Cr6 steel ball wear traces after tribological testing with PLA CF at 30% infill, following the removal of adhered PLA CF debris; (**d**) PLA CF specimen with 100% gyroid pattern infill wear traces; (**e**) 100Cr6 steel ball covered with PLA CF debris after tribological testing of 100% infill specimen; (**f**) 100Cr6 steel ball wear traces after tribological testing with PLA CF at 100% infill, following the removal of adhered PLA CF debris.

**Figure 11:**
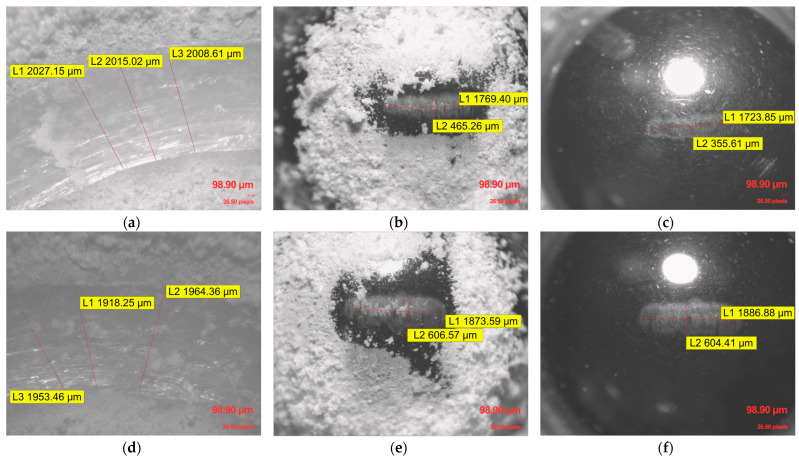
Microscopic evaluation of the wear track morphology and counterbody surface after Ball-on-Disc testing of ApolloX Kevlar material: (**a**) ApolloX Kevlar specimen with 10% gyroid pattern infill wear traces; (**b**) 100Cr6 steel ball covered with ApolloX Kevlar debris after tribological testing of 10% infill specimen; (**c**) 100Cr6 steel ball wear traces after tribological testing with ApolloX Kevlar at 10% infill, following the removal of adhered ApolloX Kevlar residues; (**d**) ApolloX Kevlar specimen with 90% gyroid pattern infill wear traces; (**e**) 100Cr6 steel ball covered with ApolloX Kevlar debris after tribological testing of 90% infill specimen; (**f**) 100Cr6 steel ball wear traces after tribological testing with ApolloX Kevlar at 90% infill, following the removal of adhered ApolloX Kevlar residues.

This consistent macro-texture suggests that the chosen printing parameters remained uniform, creating a reproducible initial surface state for the Ball-on-Disc tribological evaluation. Within the entire set of examined infill density specimens and materials, the Archimedean top pattern plays a crucial role in the initial contact mechanics. These raised ridges likely serve as primary contact points, leading to initial wear concentrated on these features. Significant accumulation of wear debris, appearing as fine particulates and larger agglomerates, was observed within and adjacent to the wear on generic PLA specimens (Figure 9b,c), PLA CF specimens (Figure 10a,b,d,e), and ApolloX Kevlar specimens (Figure 11a,b,d,e) specimens, indicating continuous material removal during sliding.

The inherent gyroid layered structure resulting from the FDM process was generally not affected. In the case of these wear-affected materials, the 100Cr6 steel ball created a channel within the materials, which led to the degradation of the specimen’s upper layers, specifically, the alteration of the Archimedean top pattern. As observed in Figure 10c,f and Figure 11c,f, even the 100Cr6 steel counterbody exhibited wear marks due to the abrasive action of the PLA CF and Kevlar-based materials.

For the specimens fabricated with Iglidur I180-BL tribofilament, no visible wear marks were observed on the analyzed surfaces. Only in the case of the specimen with 10% infill density was a fine gray powder detected on the surface of the testing steel ball. In the case of Iglidur I180-BL, the top layer remained unaltered and the Archimedean pattern was preserved, which suggests the material’s excellent wear resistance, making it ideal for applications where wear resistance is a critical factor.

### 3.4. Shore Hardness Evaluation Results

The Shore D hardness values generally exhibited an increasing trend with greater infill density for all tested materials.

Generic PLA specimens steadily demonstrated the highest Shore D hardness values across all infill densities, ranging from 76 Shore D for 10% gyroid infill to a peak of 85 Shore D for 70% gyroid infill. The hardness of generic PLA showed a gradual increase from 10% to 50% infill, followed by a relatively constant region at higher infill densities. The Iglidur I180-BL tribofilament specimens consistently exhibited the lowest Shore D hardness values, starting at 43 Shore D for 10% infill and reaching a maximum of 71 Shore D for 90% and 70 Shore D for 100% infill. For Iglidur I180-BL, a considerable increase in hardness occurred between 10% and 30% infill density, followed by a more gradual increase that stabilized at the highest infill densities.

The PLA CF and ApolloX Kevlar composites did not result in higher Shore D hardness compared to generic PLA. At equivalent infill densities, both reinforced materials showed lower hardness values than generic PLA. For the 100% infill specimens, PLA CF (81 Shore D) and ApolloX Kevlar (74 Shore D) were notably softer than generic PLA (84.5 Shore D). PLA CF demonstrated an increasing trend in hardness with infill, from 51 Shore D at 10% to 81 Shore D at 100% infill. ApolloX Kevlar also showed an increasing trend, from 61 Shore D at 10% infill to 74 Shore D at 100% infill densities, with stabilization observed between 70% and 90% gyroid infill. Table 10 presents the average recorded Shore hardness values for all studied materials and all gyroid pattern infill densities.

### 3.5. Comparative Materials Performance Results

A comprehensive comparison of the materials studied reveals distinct tribological profiles:

- Lowest Friction and Wear: Iglidur I180-BL stands out as the best performer, consistently achieving very low friction coefficients and exhibiting virtually no visible wear or material transfer to the 100Cr6 steel ball. This is attributed to its specialized composition designed for self-lubrication. The PLA 90% infill density sample also exhibited exceptional low friction and minimal wear through a fine polishing mechanism, suggesting that optimized infill can significantly improve the tribological response of generic PLA.
- Moderate Friction, Variable Wear: PLA samples generally showed moderate friction (average ~0.4) with varying degrees of wear and debris formation, including laminar chips for lower infills and a relatively deep profile for 50% infill specimens. This variability underscores the importance of infill density in controlling PLA’s wear behavior.
- Moderate Friction, Visible Wear: PLA CF and ApolloX Kevlar specimens both exhibited moderate friction coefficients. However, they demonstrated more pronounced wear compared to Iglidur I180-BL. ApolloX Kevlar filament consistently resulted in deep profiles, significant debris, and clear wear scars on the steel ball, as evidenced by microscopic images and measurements. This suggests that while fiber reinforcement enhances bulk mechanical properties, it can lead to increased abrasive wear under sliding conditions if the fibers become exposed or detached, acting as hard particles that abrade both the polymer matrix and the counter-surface, in this case, the 100Cr6 steel ball. PLA CF also showed visible profiles and ball traces for most infills, indicating similar abrasive mechanisms from carbon fibers.

The experimental tribological results align well with existing literature for comparable FDM materials. For PLA, typical COF values range from 0.35 to 0.55 [15,35], consistent with our experimental data. The literature also reports PLA COF values as low as 0.04 under lubrication and up to 0.3 in dry sliding [36,37]. PLA’s average linear wear is typically 15.2 µm/km [31]. Carbon fiber-reinforced PLA consistently shows a reduced specific wear rate, up to 70%, and lower COF compared to neat PLA [38,39], validating our results. Iglidur I180 similarly exhibits a COF of approximately 0.278 and immeasurable wear [39]. While PTFE/Kevlar fabric liners are not FDM-fabricated, studies confirm their enhanced performance with reinforcements, showing 7.6–11.8% COF reduction and 36.7–58.8% wear volume decrease [40].

To investigate the relationship between Shore D hardness and tribological performance, Pearson correlation coefficients were calculated across all material types and infill densities. A strong negative correlation (r = −0.81) was observed between Shore D hardness and wear depth, indicating that increased hardness generally contributed to improved wear resistance. A moderate negative correlation (r = −0.62) was also found between Shore D hardness and the average coefficient of friction (COF), suggesting that stiffer surfaces may offer lower contact area deformation and reduced friction during sliding.

These trends were especially evident for generic PLA and PLA CF specimens, where higher infill density resulted in increased Shore D values and simultaneously decreased wear track depth and COF. Exceptions were noted for Kevlar-based specimens, where the abrasive wear mechanism dominated regardless of Shore hardness.

**Figure 12:**
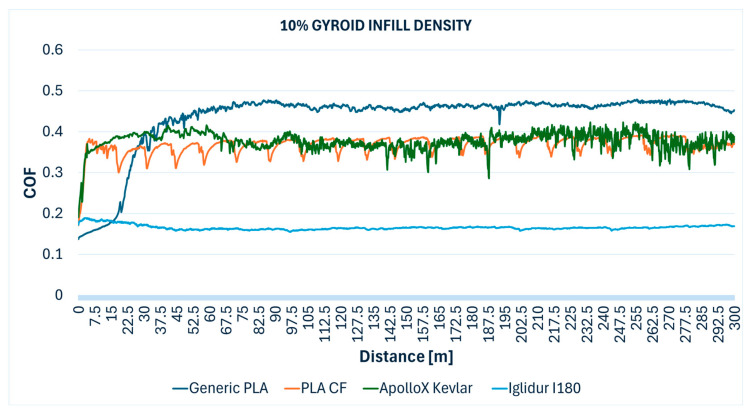
Friction coefficient comparative evolution of the 10% gyroid infill specimens during Ball-on-Disc test.

**Figure 13:**
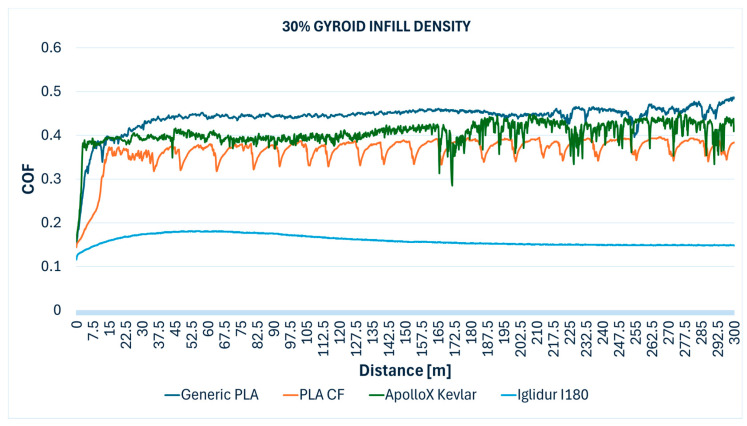
Friction coefficient comparative evolution of the 30% gyroid infill specimens during Ball-on-Disc test.

**Figure 14:**
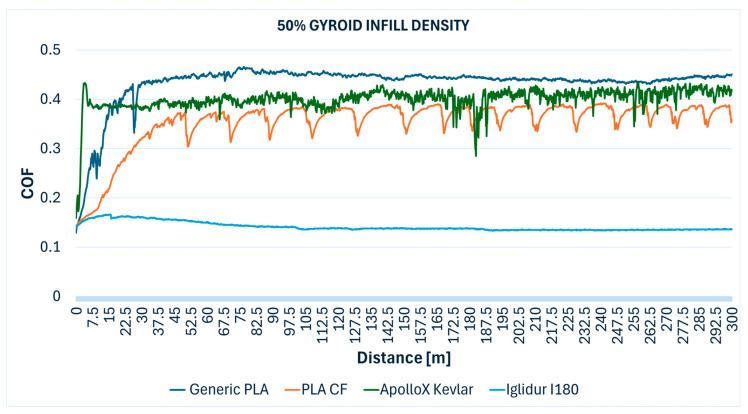
Friction coefficient comparative evolution of the 50% gyroid infill specimens during Ball-on-Disc test.

**Figure 15:**
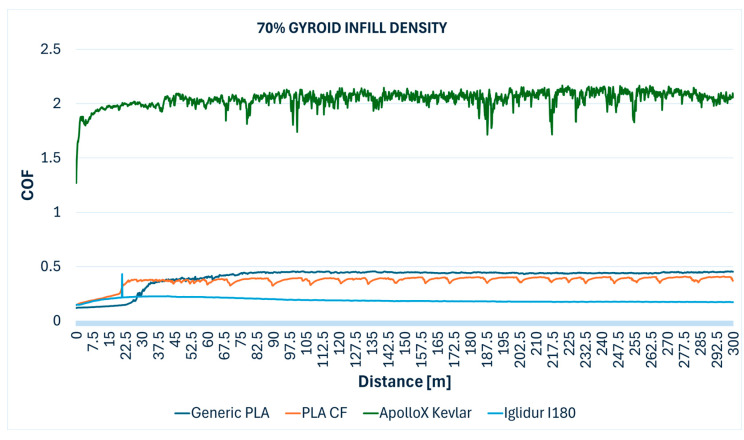
Friction coefficient comparative evolution of the 70% gyroid infill specimens during Ball-on-Disc test.

**Figure 16:**
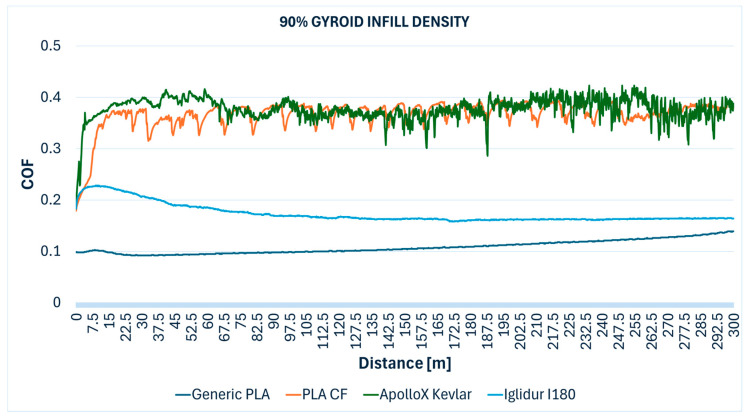
Friction coefficient comparative evolution of the 90% gyroid infill specimens during Ball-on-Disc test.

**Figure 17:**
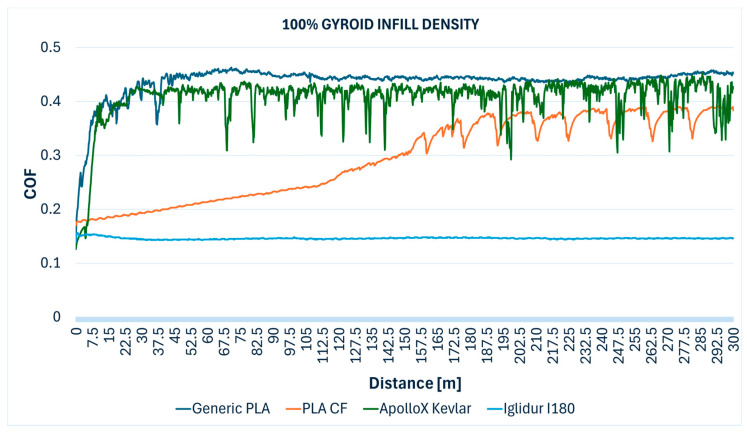
Friction coefficient comparative evolution of the 100% gyroid infill specimens during Ball-on-Disc test.

The comparative evolution of the COF measured during the Ball-on-Disc testing of the specimens with different gyroid infill densities are presented below: 10% (Figure 12), 30% (Figure 13), 50% (Figure 14), 70% (Figure 15), 90% (Figure 16), and 100% (Figure 17).

**Figure 18:**
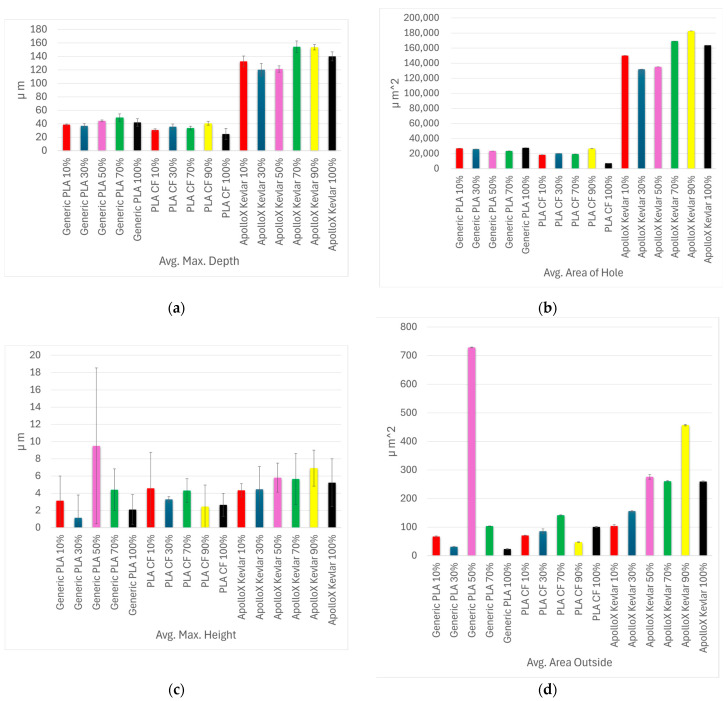
Comparative analysis of surface wear for specimens exhibiting a wear track left by the testing ball: (**a**) average maximum wear depth; (**b**) average maximum wear height; (**c**) average area of wear hole; (**d**) average area outside.

Figure 18 shows a comparative analysis of the specimens that exhibited pronounced wear marks following tribological testing, with a focus on the following key surface parameters: average maximum depth (Figure 18a), average maximum height (Figure 18b), average area of the wear hole (Figure 18c), and average area outside the wear track (Figure 18d).

**Figure 19:**
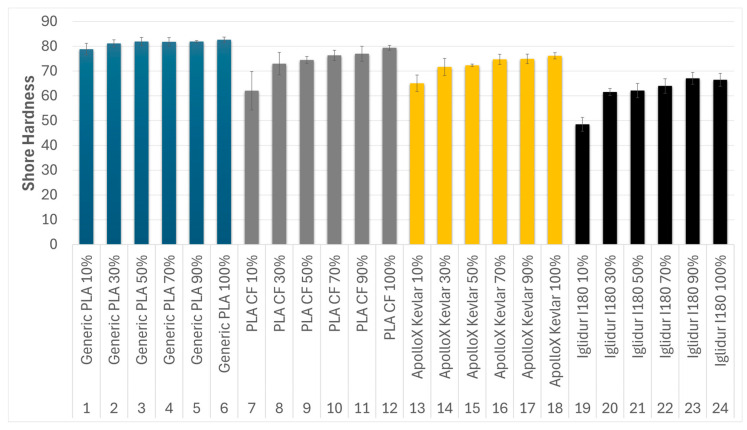
Comparative Shore hardness of studied specimens at different infill percentages.

Figure 19 provides a comparative overview of the Shore D hardness values measured for all studied specimens across different gyroid infill densities. This graphical representation highlights the influence of both material type and infill density on the surface hardness response, enabling direct comparison of mechanical performance under varying the gyroid structure. The observed increase in Shore D hardness for specimens with denser gyroid infill structures can be explained by the enhanced internal support these patterns provide. Denser and interconnected geometries better resist localized deformation from the Shore durometer tip, resulting in higher recorded hardness values.

To validate the observed relationship between hardness and tribological behavior, a Pearson correlation coefficient was calculated between Shore D hardness and average wear track depth across all specimens. The resulting value of r = −0.84 indicates a strong negative correlation, confirming that materials with higher hardness tend to exhibit lower wear depth under identical testing conditions.

## 4. Discussion

### 4.1. Effect of Top Archimedean Pattern Surface

The FDM-printed specimens exhibit a distinct outer surface characteristic, particularly evident in the microscopic images, which display a curvilinear pattern consistent with an Archimedean chord toolpath for shell layers. This layered topography, with its inherent ridges and valleys, significantly influences the initial contact mechanics and subsequent sliding behavior. Initially, contact between the steel ball and the polymer surface likely occurs at the high points or ridges of this surface. This localized contact can lead to high initial contact stresses, potentially causing immediate localized plastic deformation or fracture of these asperities. The deep and visible profile observations of ApolloX Kevlar and PLA suggest significant deformation and material removal conforming to the shape of the sliding ball. The anisotropic nature of the FDM-printed pattern (e.g., layers oriented perpendicular to the sliding direction) could lead to anisotropic wear. As sliding progresses, these surface features are worn down, generating wear debris. This debris can become entrapped in the valleys of the texture or between the sliding surfaces, potentially acting as a third-body abrasive, increasing friction variability, and contributing to further material removal. The initial contact conformity might be low due to the rough texture, but as wear progresses, a larger, smoother contact area may form within the wear track, potentially stabilizing friction. However, the continuous generation and entrapment of debris from the structured surface can also lead to stick–slip phenomena or erratic friction.

### 4.2. Analysis of the Effect of Gyroid Infill Density

The gyroid internal pattern structure plays a fundamental role in determining structural stiffness, damping characteristics, and the overall mechanical response of the specimens under tribological load. Higher infill densities (90%–100%) result in more rigid and continuous internal frameworks, supporting surface layers and distributing applied loads more evenly. This leads to improved wear resistance and lower friction, as evidenced by the superior performance of generic PLA at 90% infill and Iglidur I180-BL across all densities. Conversely, lower infill densities (10%–30%) may allow local deformation and stress concentration, promoting material fatigue and greater wear depths, especially in PLA CF and ApolloX Kevlar.

The influence of structural geometry on tribological performance has been widely discussed since the early theoretical models of plastic deformation and surface energy dissipation [3,6]. The gyroid infill pattern used in this study draws on periodic minimal surfaces theory, whose application to lightweight, load-bearing structures was first explored in the aerospace and biomedical fields [7,8]. Recent experimental validations of these theoretical assumptions in polymer matrix composites further support the idea that internal architecture governs load transfer and contact stress distribution [6,9].

The analysis revealed that the COF of PLA materials decreased with increasing infill, indicating a reduction in friction due to enhanced internal support and minimized surface deflection. However, for Kevlar and PLA CF, which contain reinforcing fibers, infill density did not significantly alter friction behavior, likely because the wear mechanism is dominated by fiber–matrix interactions rather than bulk geometry. These observations suggest that for fiber-reinforced composites, surface characteristics and filler orientation may overshadow the influence of internal architecture.

Higher infill densities, particularly with a gyroid pattern, enhance structural rigidity and constrain polymer chain mobility, reducing local deformation under Shore D indentation and leading to higher hardness values.

### 4.3. Proposed Wear Mechanisms

The observed wear behaviors suggest distinct mechanisms across materials:

- Plastic deformation and abrasive wear: Evident in ApolloX Kevlar and PLA CF, where detachment of hard fibers (Kevlar or carbon) likely contributed to abrasive third-body wear. These materials exhibited deep profiles, visible wear debris, and steel ball scarring.
- Material transfer (adhesive wear): The presence of adhered polymer material on the 100Cr6 steel ball in PLA CF and Kevlar indicates adhesive wear, wherein material is sheared from the surface and transferred to the counterface.
- Surface polishing: For high-infill PLA, notably 90%, wear manifested as polishing rather than material loss, indicating mild abrasive contact and uniform stress distribution.
- Self-lubrication: Iglidur I180-BL’s performance confirms the presence of solid lubricants in its matrix. The absence of significant wear or debris supports a wear regime dominated by cohesive shearing and reduced interfacial shear strength.

### 4.4. Quantitative Performance Comparisons

To consolidate findings, it is useful to compare performance metrics across materials:

- Lowest COF (average): Iglidur I180-BL (0.146–0.190); PLA 90% infill (0.108).
- Greatest wear depth: ApolloX Kevlar at 90% infill (Avg. Max. Depth ~154 µm).
- Highest hardness: Generic PLA (up to 82.7 Shore D); PLA CF (79.7 Shore D).
- Surface roughness (Ra): PLA CF is generally lower than Kevlar or generic PLA, correlating with increased reinforcement but also increased abrasive effect.

These quantitative insights confirm that optimized infill (90%–100%) enhances wear resistance in unreinforced PLA, while fiber reinforcements, although improving mechanical strength, introduce abrasive wear challenges. Iglidur I180-BL consistently outperforms due to its intrinsic tribological design.

Although Iglidur I180-BL is commercially marketed as a tribologically optimized filament with a COF between 0.15 and 0.25, our experiments revealed slightly lower values for generic PLA at 90% infill (average COF ~0.108). This discrepancy can be attributed to the distinct polishing-type wear mechanism observed for high-density PLA, which resulted in a smooth, continuous wear track with minimal surface damage or friction variability. In contrast, Iglidur’s COF, although higher numerically, remained extremely stable across all densities and test durations. It is important to emphasize that the superior tribological behavior of Iglidur is not solely defined by minimum COF but by the complete absence of wear, debris, or surface transfer, confirming its design for self-lubrication and durability.

The coefficient of friction (COF) values and wear depths obtained in the present study are in strong agreement with existing literature. For instance, previous investigations reported COF values in the range of 0.09–0.12 for PLA reinforced with short carbon fibers and graphene nanoplatelets, which closely aligns with the COF of approximately 0.108 observed here for PLA specimens with 90% infill [3]. Similarly, average COF values of 0.16–0.21 have been reported for PLA–aluminum composites under dry sliding conditions, corroborating the tribological performance exhibited by PLA CF specimens in this work [8]. Regarding wear behavior, standard PLA samples have shown wear depths of up to 120 µm under comparable testing conditions, consistent with the maximum wear depths measured in the present study for Kevlar- and carbon fiber-reinforced PLA composites [21]. These correlations reinforce the validity of the experimental results and emphasize the significance of infill optimization as an effective design strategy for reducing friction and enhancing wear resistance in FDM-printed polymers.

The tribological trends observed in this study are further supported by prior findings. Notably, PLA composites incorporating short carbon fibers and graphene nanoplatelets have been shown to exhibit considerably lower wear rates, albeit with increased surface hardness and the potential for abrasive contact mechanisms—trends mirrored by the PLA CF samples analyzed here [11]. Similarly, enhancements in hardness exceeding 75% and significant reductions in wear volume have been documented in PLA composites reinforced with metallic aluminum particles, highlighting the critical role of structural stiffness in wear mitigation [12].

The low COF values recorded for high-infill PLA specimens in this work are also consistent with previously reported near-superlubricity effects observed in dry-sliding tests of textured PLA surfaces [11]. These results underscore the synergistic benefits of combining internal mesostructural control with engineered surface texturing. Altogether, the agreement between current findings and published data substantiates the experimental methodology and confirms the efficacy of tailored infill designs for improving the tribological performance of FDM-fabricated polymer components.

The average coefficient of friction (COF) obtained for PLA in this study, particularly at 90% infill (0.108), aligns with previous studies such as [36], which reported values ranging from 0.11 to 0.16 depending on infill pattern and applied load conditions, confirming the consistency of our data within the expected performance range. Similarly, the wear depth values reported in this study agree with the findings of [15,40], confirming the relevance of infill optimization for improving wear resistance.

These comparative results support the robustness of our methodology and indicate good reproducibility with existing data.

### 4.5. Limitations and Future Work

While the present study offers robust comparative insights into the synergistic effects of infill density and surface texture on tribological and mechanical performance, several limitations should be acknowledged. First, all tribological tests were conducted under dry sliding conditions, ambient temperature (24 ± 1 °C), and constant normal load (5 N), using a single counterface material (100Cr6 steel). These conditions do not fully replicate the wide range of thermal, chemical, and mechanical environments encountered in real-world applications such as gear housings, robotic joints, or biomedical sliding interfaces. Second, the study exclusively evaluated circular disc specimens with a fixed Archimedean surface pattern and gyroid infill geometry. Other common infill structures (e.g., cubic, honeycomb, and triangular) or texturing strategies (e.g., dimples, grooves, and stochastic textures) may yield different wear and friction behaviors. Third, long-term durability under oscillating or cyclic loads, temperature fluctuations, and lubricated conditions was not addressed. The degradation mechanisms (e.g., fabric wear, thermal softening, and delamination) remain unexplored. To overcome these limitations and extend the current findings, future research should focus on:

- Evaluating the effect of lubrication (oil- or water-based), temperature gradients, and varying humidity levels on wear behavior.
- Expanding the test matrix to include different infill geometries and surface materials (e.g., aluminum, polymers, and ceramics).
- Investigating fatigue resistance and wear progression over extended sliding distances (beyond 300 m).
- Performing finite element simulations to correlate internal stress distributions with observed wear patterns.
- Exploring hybrid reinforcement strategies (e.g., combining fibers and solid lubricants) for dual enhancement of mechanical and tribological properties.

These future directions will help bridge the gap between laboratory testing and industrial application, enabling the deployment of optimized FDM components in high-performance environments.

## 5. Conclusions

The present research provides a systematic and integrative assessment of how the internal architecture (gyroid infill density) and external surface features (Archimedean pattern) jointly affect the mechanical and tribological performance of FDM-printed polymer specimens. Unlike previous studies that treat these design factors separately, our work offers a comparative, cross-material analysis involving generic PLA, PLA reinforced with carbon fibers (PLA CF), Kevlar-based ASA, and the tribologically optimized Iglidur I180-BL. Through this multivariable approach, clear performance hierarchies have been established in terms of friction, wear morphology, and hardness, contributing novel insights for design optimization in load-bearing polymeric components.

Iglidur I180-BL consistently demonstrated superior tribological performance, exhibiting exceptionally low friction and minimal to no discernible wear or material transfer to the steel counter-surface, attributed to its engineered characteristics. Generic PLA, particularly at higher infill densities, also displayed favorable low-friction behavior, with wear occurring predominantly through a polishing mechanism. By comparison, PLA CF and ApolloX Kevlar exhibited higher COF and more pronounced abrasive wear, leading to significant material removal and wear traces on the 100Cr6 steel ball, indicating that exposed reinforcing fibers contributed to an abrasive wear mechanism.

These findings offer valuable insights into the functional design and structural optimization of FDM-printed components and highlight that careful material selection, coupled with the judicious control of internal architecture and surface engineering, is mandatory to achieve desired tribological and mechanical performance in demanding applications. Future research could further explore the tribological behavior of these materials under varying environmental conditions or dynamic loading scenarios to investigate the interplay of other infill patterns and surface textures.

## Figures and Tables

**Table 1:** Comparative physical and mechanical properties of Fused Deposition Modeling (FDM)-printable polymers according to manufacturer data sheets.

Material Property.	PLA	PLA CF	Kevlar	Iglidur I180-BL	References
Material density	1.24 g/cm^3^	1.29 g/cm^3^	1.07 g/cm^3^	1.21 g/cm^3^	[19,20,21,22]
Flexural Strength	83 MPa	X–Y: 54.2 ± 1.4 MPa	N/A	33–44 MPa **	[19,20,22]
Flexural Modulus	3.8 GPa	X–Y: 3215 ± 182 MPa	N/A	1 GPa	[19,20,22]
Tensile Strength	N/A *	X–Y: 28.28 ± 0.7 MPa Z: 12.54 ± 0.7 MPa	N/A	N/A	[20]
Tensile Strength at break	53 MPa	N/A	35 MPa	N/A	[19,21]
Tensile Modulus	3.6 GPa	X–Y: 2945 ± 100 MPa Z: 2143 ± 91 MPa	2.2 GPa	N/A	[19,20,21]
Tensile Yield Strength	60 MPa	N/A	40 MPa	N/A	[19,21]
Tensile Elongation	6%	N/A	N/A	N/A	[19]
Elongation at Break	N/A	X–Y: 4.2 ± 0.12% Z: 0.75 ± 0.08%	6%	N/A	[20,21]

* N/A Value not available on manufacturer data sheet. ** It depends directly on the printing parameters, according to the technical data sheet.

**Table 2:** Specimens’ FDM printing parameters of the polymeric materials.

Printing Parameter *	PLA Generic	PLA CF	ApolloX Kevlar	Iglidur I180-BL
Print Temperature	220 °C	220 °C	255 °C	260 °C
Hot Pad	60 °C	60 °C	90 °C	90 °C
Printing Speed	60 mm/s	60 mm/s	25 mm/s	20 mm/s
Travel Speed	500 mm/s	500 mm/s	300 mm/s	50 mm/s

* All printing parameters were selected based on preliminary optimization trials and/or manufacturer data sheets and are tailored to ensure dimensional accuracy and material integrity during FDM printing on a Creality K1C system.

**Table 3:** Summary of friction coefficient measurements of the generic PLA (polylactic acid) specimens.

Infill Density [%]	COF Average * ± SD **	Min. COF	Max. COF
10	0.435 ± 0.025	0.137	0.479
30	0.441 ± 0.018	0.150	0.487
50	0.433 ± 0.027	0.129	0.166
70	0.406 ± 0.022	0.118	0.457
90	0.108 ± 0.009	0.092	0.139
100	0.437 ± 0.020	0.146	0.463

* The average coefficient of friction was calculated using Equation (1). ** The standard deviation (SD) was calculated using Equation (2).

**Table 4:** Summary of friction coefficient measurements of the PLA CF polylactic acid with carbon fiber) specimens.

Infill Density [%]	COF Average * ± SD **	Min. COF	Max. COF
10	0.367 ± 0.055	0.171	0.391
30	0.365 ± 0.063	0.144	0.396
50	0.355 ± 0.063	0.139	0.392
70	0.370 ± 0.066	0.143	0.409
90	0.367 ± 0.054	0.179	0.395
100	0.296 ± 0.056	0.170	0.392

* The average coefficient of friction was calculated using Equation (1). ** The standard deviation (SD) was calculated using Equation (2).

**Table 5:** Summary of friction coefficient measurements of the ApolloX Kevlar specimens.

Infill Density [%]	COF Average * ± SD **	Min. COF	Max. COF
10	0.377 ± 0.058	0.191	0.423
30	0.404 ± 0.073	0.158	0.451
50	0.400 ± 0.069	0.159	0.436
70	0.408 ± 0.045	0.254	0.433
90	0.419 ± 0.068	0.185	0.456
100	0.407 ± 0.082	0.126	0.452

* The average coefficient of friction was calculated using Equation (1). ** The standard deviation (SD) was calculated using Equation (2).

**Table 6:** Summary of friction coefficient measurements of the Iglidur I180-BL specimens.

Infill Density [%]	COF Average * ± SD **	Min. COF	Max. COF
10	0.166 ± 0.008	0.155	0.189
30	0.160 ± 0.016	0.116	0.181
50	0.141 ± 0.008	0.133	0.166
70	0.190 ± 0.071	0.146	0.431
90	0.173 ± 0.018	0.158	0.228
100	0.146 ± 0.004	0.140	0.156

* The average coefficient of friction was calculated using Equation (1). ** The standard deviation (SD) was calculated using Equation (2).

**Table 7:** Average wear track measurements for generic PLA specimens at different infill densities.

Infill Density [%]	Avg. Max. Depth * [µm] ± SD **	Avg. Area of the Hole *^,^*** [µm^2^] ± SD **	Avg. Max. Height * [µm] ± SD **	Avg. Area Outside * [µm^2^] ± SD **
10	38.82 ± 0.89	26,965 ± 8.24	3.13 ± 2.87	67.11 ± 2.13
30	36.87 ± 3.44	25,994 ± 3.15	1.13 ± 2.65	31.45 ± 1.67
50	44.23 ± 1.32	23,458 ± 0.004	9.49 ± 9.04	728.66 ± 1.11
70	48.83 ± 5.80	23,511 ± 4.54	4.39 ± 2.43	103.46 ± 1.01
100	41.94 ± 5.67	27,654 ± 7.57	2.10 ± 1.73	23.25 ± 1.59

* The average values were calculated using Equation (1). ** The standard deviation (SD) was calculated using Equation (2). *** Reported values were rounded according to the profilometer’s precision. Calculations used full-resolution data.

**Table 8:** Average wear track measurements for PLA CF specimens at different infill densities.

Infill Density [%]	Avg. Max. Depth * [µm] ± SD **	Avg. Area of the Hole *^,^*** [µm^2^] ± SD **	Avg. Max. Height * [µm] ± SD **	Avg. Area Outside * [µm^2^] ± SD **
10	30.65 ± 1.87	18,381 ± 8.16	4.56 ± 4.17	70.54 ± 1.49
30	35.70 ± 4.07	20,351 ± 1.83	3.28 ± 0.32	85.64 ±8.45
70	33.68 ± 2.79	19,356 ± 1.33	4.29 ± 1.39	141.70 ± 1.59
90	40.64 ± 2.90	26,707 ± 3.31	2.43 ± 2.51	47.366 ± 1.14
100	24.83 ± 8.25	7148 ± 6.85	2.64 ± 1.33	100.59 ± 2.41

* The average values were calculated using Equation (1). ** The standard deviation (SD) was calculated using Equation (2). *** Reported values were rounded according to the profilometer’s precision. Calculations used full-resolution data.

**Table 9:** Average wear track measurements for ApolloX Kevlar specimens at different infill densities.

Infill Density. [%]	Avg. Max. Depth * [µm] ± SD **	Avg. Area of the Hole *^,^*** [µm^2^] ± SD **	Avg. Max. Height * [µm] ± SD **	Avg. Area Outside * [µm^2^] ± SD **
10	132.66 ± 7.89	150,103 ± 0.017	4.33 ± 0.77	104.06 ± 4.47
30	120.36 ± 9.16	131,897 ± 0.03	4.46 ± 2.63	156.03 ± 1.74
50	121.33 ± 4.72	135,091 ± 0.009	5.81 ± 1.68	276.00 ± 8.11
70	154.33 ± 8.63	169,201 ± 0.031	5.64 ± 2.97	260.80 ± 2.18
90	153.66 ± 4.04	182,667 ± 0.004	6.91 ± 2.08	457.00 ± 2.37
100	140.00 ± 6.68	163,708 ± 0.022	5.22 ± 2.77	259.66 ± 2.16

* The average values were calculated using Equation (1). ** The standard deviation (SD) was calculated using Equation (2). *** Reported values were rounded according to the profilometer’s precision. Calculations used full-resolution data.

**Table 10:** Shore hardness analysis of FDM polymeric specimens.

Material	Infill Density [%]	Avg. Shore Hardness * ± SD **
Generic PLA	10 30 50 70 90 100	78.90 ± 2.33 81.20 ± 1.43 82.00 ± 1.68 81.70 ± 1.72 81.80 ± 0.37 82.70 ± 1.03
PLA CF	10 30 50 70 90 100	64.30 ± 7.76 74.10 ± 4.53 74.80 ± 1.34 76.90 ± 2.06 76.80 ± 3.04 79.70 ± 1.02
ApolloX Kevlar	10 30 50 70 90 100	65.90 ± 3.33 71.90 ± 3.45 72.30 ± 0.51 75.00 ± 2.04 75.10 ± 1.93 71.50 ± 1.29
Iglidur I180-BL	10 30 50 70 90 100	48.10 ± 2.79 61.90 ± 1.39 62.30 ± 2.89 64.40 ± 2.91 67.30 ± 2.37 67.30 ± 2.64

* The average values were calculated using Equation (1). ** The standard deviation (SD) was calculated using Equation (2).

## Data Availability

Data is contained within the article.
